# On the Normalization of the Minimum Free Energy of RNAs by Sequence Length

**DOI:** 10.1371/journal.pone.0113380

**Published:** 2014-11-18

**Authors:** Edoardo Trotta

**Affiliations:** Institute of Translational Pharmacology, Consiglio Nazionale delle Ricerche (CNR), Roma, Italy; Ben-Gurion University, Israel

## Abstract

The minimum free energy (MFE) of ribonucleic acids (RNAs) increases at an apparent linear rate with sequence length. Simple indices, obtained by dividing the MFE by the number of nucleotides, have been used for a direct comparison of the folding stability of RNAs of various sizes. Although this normalization procedure has been used in several studies, the relationship between normalized MFE and length has not yet been investigated in detail. Here, we demonstrate that the variation of MFE with sequence length is not linear and is significantly biased by the mathematical formula used for the normalization procedure. For this reason, the normalized MFEs strongly decrease as hyperbolic functions of length and produce unreliable results when applied for the comparison of sequences with different sizes. We also propose a simple modification of the normalization formula that corrects the bias enabling the use of the normalized MFE for RNAs longer than 40 nt. Using the new corrected normalized index, we analyzed the folding free energies of different human RNA families showing that most of them present an average MFE density more negative than expected for a typical genomic sequence. Furthermore, we found that a well-defined and restricted range of MFE density characterizes each RNA family, suggesting the use of our corrected normalized index to improve RNA prediction algorithms. Finally, in coding and functional human RNAs the MFE density appears scarcely correlated with sequence length, consistent with a negligible role of thermodynamic stability demands in determining RNA size.

## Introduction

The cell synthesizes various types of RNAs that play distinctive and essential roles in living systems, including coding (mRNA), decoding (tRNA), catalytic (ribozymes), regulatory (e.g., microRNA), and structural (e.g., rRNA) functions. The cellular activity of each RNA is normally dependent on the specific structural features of its functional category. This critical role of structure in the function of RNA molecules, together with its difficulty in being determined experimentally [1], have favoured the development of a number of software packages that predict RNA secondary structure. These include computer programs based on minimum free energy (MFE) algorithms. The MFE of an RNA molecule is affected by three properties of nucleotides in the sequence: their number, composition, and arrangement. In fact, longer sequences are on average more stable because they can form more stacking and hydrogen bond interactions, guanine-cytosine (GC)-rich RNAs are typically more stable than adenine-uracil (AU)-rich sequences, and nucleotide order influences the folding structure stability because it determines the number and the extension of loops and double-helix conformations. It has been found that mRNAs and microRNA precursors, unlike other non-coding RNAs, have greater negative MFE than expected given their nucleotide numbers and compositions [2], [3]. This led to the observation that free energy can be employed as a criterion for the identification of functional RNAs. However, when the folding energies of different classes of RNA are compared, the dependence of MFE to sequence length can represent a disturbing element. To overcome this obstacle, a new class of free energy indices normalized by sequence length has been proposed. These indices can be conceived as free energy density indicators and were obtained simply by dividing MFE by the number of nucleotides in the sequence [4]–[9]. A widely used normalized index is the so-called adjusted MFE (AMFE) [9]. AMFE is calculated by dividing MFE by the sequence length and then multiplying the result by 100 to relate the index to a segment of 100 nucleotides. Based on their supposed weak relationship with sequence length, normalized MFEs have been used in a number of published works to compare the free energy among different classes of RNAs. In fact, it has been reported that, after this adjustment, the MFEs of all nucleotide sequences are comparable [9]. Furthermore, it was also reported that length-normalization renders the MFE of hairpins of different lengths comparable [6] and provides an estimate of stability that is not influenced by differences in RNA sequence length [10]. However, even if the length-normalized MFEs have been used in a number of studies, to our knowledge, their relationship with sequence size has not been thoroughly tested and lacks quantitative substantiation. Using simulated sequences, we searched for possible residual components of AMFE associated with length. We found that the suggested procedure for normalizing MFE by length produces unacceptable results. AMFE is significantly affected by sequence length, leading to substantial errors if the index is used directly to compare the stability of RNA sequences of various lengths. We show that the error is generated by the combined effects of a poor mathematical normalization procedure and a non-perfect linear relationship between MFE and sequence length. To allow the direct comparison of the MFE of differently sized RNAs, here we propose a correction in the normalization procedure that removes the AMFE bias extending its applicability to all RNAs longer than 40 nt. Using the new normalized index, termed MFE density (MFEden), we report the analysis of a set of human coding and functional RNA families.

## Results

### Comparative software analysis

The most common software programs, employed to predict the secondary RNA structures by MFE algorithms, make use of the so-called nearest-neighbor energy model. This model uses free energy rules based on empirical thermodynamic parameters [11], [12] and computes the overall stability of an RNA structure by adding independent contributions of local free energy interactions due to adjacent base pairs and loop regions. In sequences with homogeneous nucleotide arrangements and compositions, the additive and independent nature of the local free energy contributions suggests a linear relationship between computed MFE and sequence length. Normalization by length, obtained by dividing MFE by the number of nucleotides, was introduced to exploit this linear relationship to directly compare the minimum free energies of RNAs of various lengths. To investigate on the relationship of MFE and length-normalized MFE with sequence size, we computed MFE by two of the most common software programs used to predict RNA secondary structure through the free energy minimization approach: Quikfold application, which is incorporated in the Mfold webserver for multiple molecule processing [13], [14], and RNAfold, which is included in the ViennaRNA software package [15], [16]. The results obtained from the two programs were very similar, and the differences were irrelevant to the objective of this study. For this reason, we omitted the data from both software programs for each result.

### The relationship of length with MFE and normalized-MFE in randomly shuffled sequences containing equal frequencies of A, C, G, and U

The length-normalized index AMFE is computed by using the formula AMFE  = 100* MFE/L, where L is the number of nucleotides of the RNA sequence [9]. To determine whether sequence length affects AMFE, as an initial analysis, we generated random sequences of various lengths and equal frequencies of A, C, G, and U. Starting with a set of sequences containing one copy for each different length (from 12 to 600 nt with steps of 12 nt) and exact equal frequencies of the four bases, we generated 1000 sets of randomly shuffled sequences. Then, for each simulated length, represented by 1000 randomized sequences, we computed the mean and standard deviation (SD) values of MFE and AMFE.

**Figure 1:**
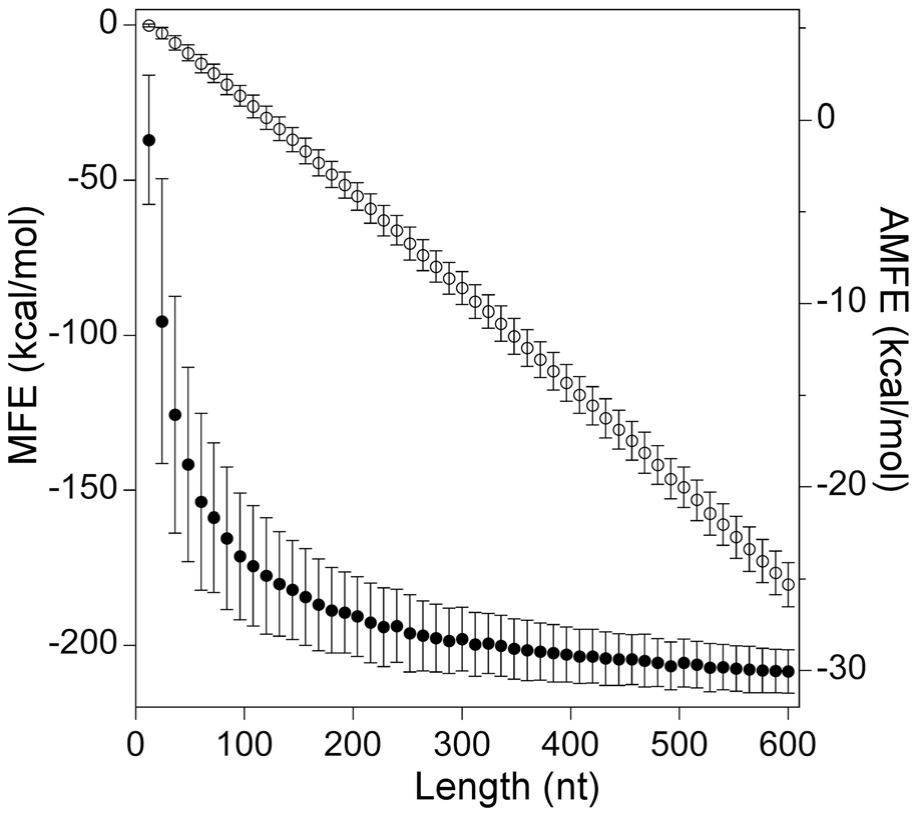
MFE and AMFE versus sequence length. For each sequence length, containing an exact equal frequency of the four nucleotides, 1000 randomly shuffled sequences were simulated. The mean values of the MFE (open circles) and AMFE (closed circles) of the shuffled sequences are plotted versus the sequence length. Vertical bars indicate standard deviations (N = 1000). MFE was computed by RNAfold using default parameters.

**Figure 2:**
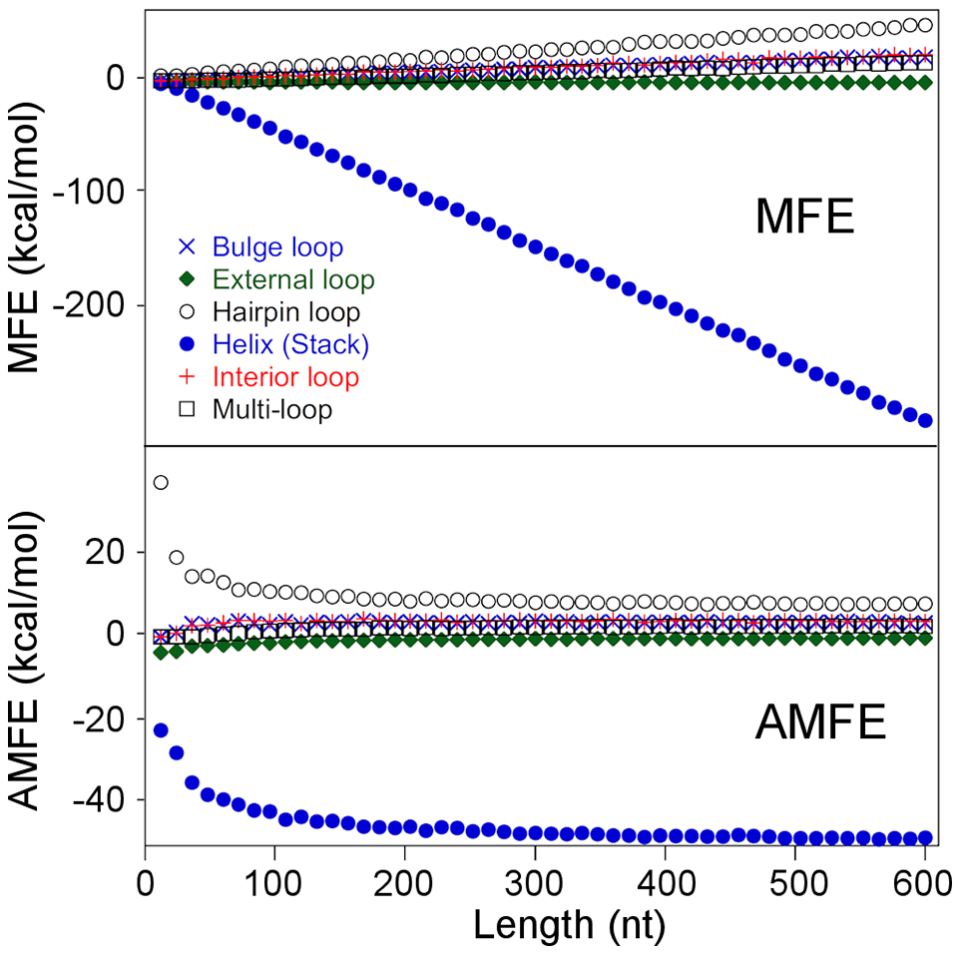
Free energy contributions of RNA structural elements. The free energy contributions of the different structural elements calculated by Quickfold are plotted versus sequence length: external loop (closed diamonds), hairpin loop (open circles), helix (closed circles), bulged loop (X), multi-loop (open squares), and interior loop (plus). The upper panel shows the contributions of structural elements to MFE and the lower panel the contributions to AMFE.

As shown by open circles in the graph in Figure 1, the increase of sequence length from 12 to 600 nt causes an apparent linear decrease of the MFE of about −180 kcal/mol at the average rate of −32 kcal/mol every 100 nucleotides. In contrast, indicated by closed circles in the graph in Figure 1, AMFE decreases, by almost 30 kcal/mol, as a hyperbolic function of length, demonstrating that a significant portion of AMFE is correlated with the sequence size.Using the RNA 3.0 (Quickfold) free energy rules [13], we computed the portion of the total minimum free energy associated with the differently classified structural elements. The upper panel in Figure 2 shows the graph of the free energy contributions of the various structural elements versus the sequence length of simulated sequences. As illustrated in the figure, base pair stacking is the most stabilizing element in our simulated sequences by a free energy contribution negatively correlated with length. Loops tend to destabilize minimum folding energy structures by quantities that, distinct from stacking energies, correlate positively with sequence length. Structural elements classified as external loops, which comprise single-stranded nucleotides and base pairs at the end of helices that are not in a loop, are weakly stabilizing and their free energy contribution decreases with length (from −1 to −1.7 kcal/mole).

The normalization by length of the individual MFE contribution from each structural element indicates that stacking and hairpin loop interactions are responsible for almost all AMFE variability associated with sequence length (Figure 2, lower panel).

**Figure 3:**
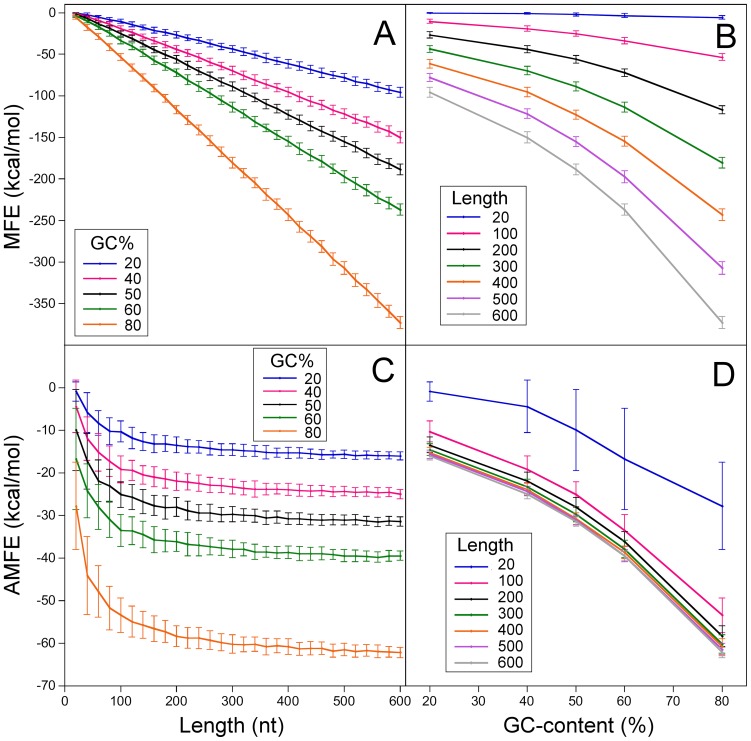
Minimum folding energy of randomly shuffled sequences. MFE (panel A) and AMFE (panel C) versus length at different GC-content: 20%, 40%, 50%, 60%, and 80%. MFE (panel B) and AMFE (panel D) versus GC-content for different sequence lengths: 20 nt, 100 nt, 200 nt, 300 nt, 400 nt, 500 nt, and 600 nt. Vertical bars indicate standard deviations (N = 100).

Comparison of the free energy variability associated with sequence length, nucleotide composition and nucleotide orderTo evaluate the impact of length to the overall variation of normalized-MFE, we should compare its effects with those generated by varying the order and the composition of nucleotides in the sequences. To this end, we generated 100 sets of randomly shuffled sequences from a set with increasing lengths and GC-contents. The length of shuffled sequences ranged between 20 and 600 nt, with steps of 20 nt. For each length, GC-contents were 20%, 40%, 50%, 60%, and 80%. The results are summarized in Figure 3, where the mean MFEs and the mean AMFEs of each randomly shuffled sequence are plotted versus length and GC-content. As illustrated in Figures 3A and 3B, the average stability of shuffled sequences increases with both length and GC-content. Increasing GC-content at constant length causes a nonlinear decrease of MFE that is more prominent for longer sequences (Figure 3B). From 20% to 80% of GC-content, the folding stability of 20 nt-long RNA increases by −5.4 kcal/mol, whereas that of 600 nt-long sequences increases by -277.0 kcal/mol.

The variation of MFE with length, at constant GC-content, is plotted in Figure 3A. The relationship between MFE and length is apparently linear, and the MFE change rate increases with GC-content. For lengths varying from 20 to 600 nt, MFE changes by about −95.5 kcal/mol in sequences with 20% of GC-content and by −367.2 kcal/mol in sequences with 80% of GC-content.

The MFE variability associated with the nucleotide arrangement in the sequence was quantified by the SD of MFE in randomly shuffled sequences (N = 100) at fixed GC-content and length. In the range analyzed (20%≤GC≤80% and 20 nt≤length≤600 nt), SD of MFE varied from 0.45 kcal/mol (GC = 20%, length = 20 nt) to 7.2 kcal/mol (GC = 80%, length = 600 nt).

The above MFE data were also used to compare the effect of length, nucleotide order, and GC-content on AMFE. Figure 3 illustrates the variations of the mean AMFE with lengths at constant GC-contents (panel C) and with GC-content at constant lengths (panel D). As shown, increasing the sequence length from 20 to 600 nt, at constant GC-content, causes an AMFE change varying from -15.1 kcal/mol – measured in sequences with the lowest GC-content (20%) – to −34.37 kcal/mol for sequences with the highest GC content (80%). The variation of AMFE with GC-content is −26.9 kcal/mol for the shortest sequences (20 nt) and −46.2 kcal/mol for the longest ones (600 nt). Moreover, the SD of AMFE for shuffled sequences at fixed lengths and GC-contents ranges from 0.83 kcal/mol (GC-content = 20%, length = 480 nt) to 11.93 kcal/mol (GC-content = 80%, length = 20 nt).

Therefore, the results show that sequence length contributes to AMFE of our simulated sequences by an amount (−15.1≤ΔAMFE≤−34.4 kcal/mol) that is comparable to that associated with the variation of nucleotide composition (−26.9≤ΔAMFE≤−46.2 kcal/mol) and with the variability of AMFE produced by a random arrangement of nucleotides in the sequences (0.83 kcal/mol≤SD≤11.93 kcal/mol). This indicates that AMFE, and generally, normalized MFEs, are biased measures of the minimum free energy, tending to decrease significantly with sequence size. These length-dependent differences in AMFE measures raise serious doubts about the validity of the normalization procedure and the reliability of the results obtained using length-normalized MFEs.

### Why normalized MFE is not independent of length

**Figure 4:**
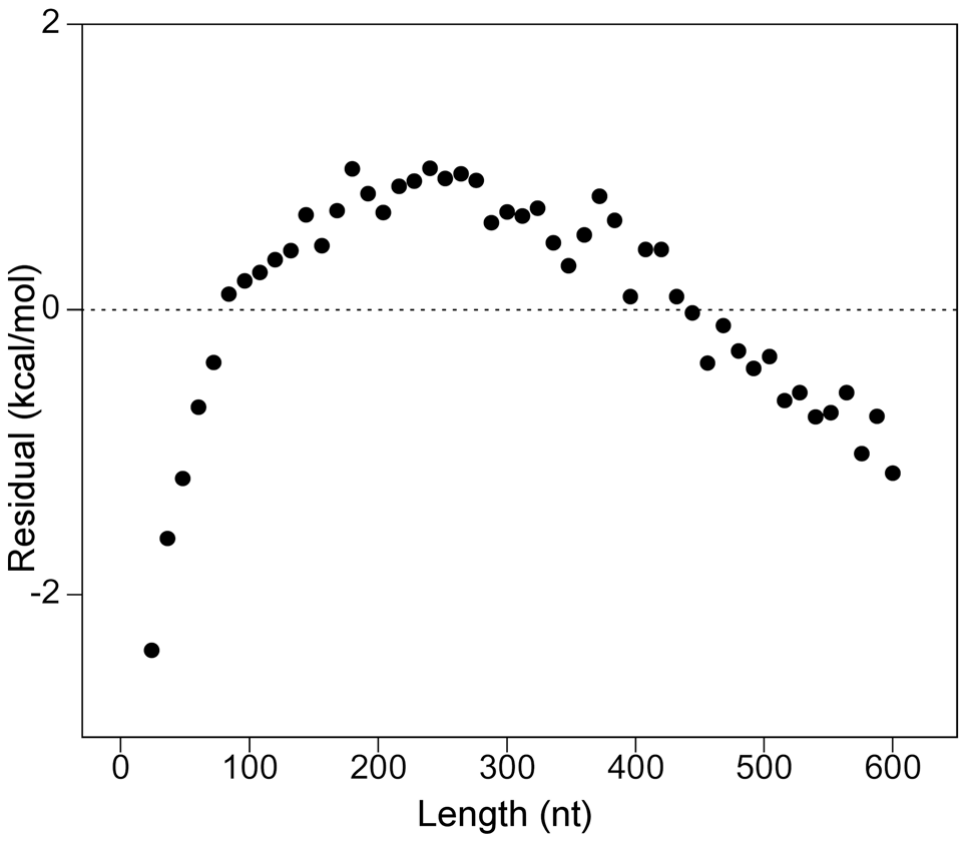
Residual plot from the linear fit of MFE versus length. Residual plot of the linear regression analysis of MFE versus sequence length. The MFE assigned to each length corresponds to the mean value of 1000 shuffled sequences with exact equimolar ratios of A, C, G, and U. Residuals are the differences between the computed MFEs and the corresponding values that are predicted by a linear regression analysis of MFEs with length.

We computed the MFE by software tools that apply the nearest-neighbor energy rules to simulate the minimum free energy secondary structure of RNA molecules. According to this model, the free energy of a structure is the result of the sum of independent contributions from various structural elements. All folded structures contain at least one destabilizing loop with a minimum length of three unpaired bases (The Nearest Neighbor Database, NNDB, http://rna.urmc.rochester.edu/NNDB) [17] and at least one base pair. Therefore, regardless of the set of energy parameters used to estimate MFE, negative free energies are not possible for sequences shorter than 5 nt. Accordingly, based on the results of the two different software programs used, the linear fitting of the MFE data versus the sequence size, at constant GC-content, intersects 0 energy axis at lengths higher than 15 nt, depending on base composition. In general, higher fitted lengths at 0 energy are associated with lower GC content. For this reason, in the case of a perfect linear relationship between MFE and length, dividing MFE by the number of nucleotides should result in a new free energy index with a hyperbolic decrease with length: if *MFE = a+b•length*, then *MFE/length = a/length+b*. Although this reason can justify the strong hyperbolic decrease of AMFE with length, this is not the only source of variability of AMFE by length. In fact, as shown by the graph in Figure 4, the residuals from a least-squared linear regression analysis of MFE versus length showed a clear pattern with length, indicating that the assumption of perfect linearity between MFE and length is not valid. In particular, the monotone concave-down curve of the residual plot in Figure 4 indicates that longer sequences tend to be more stable than expected by a linear relationship between MFE and sequence size. Consistent with this, if we translate all the MFE data by a constant amount that shifts its regression line to the origin of the graph, the ratio of the new MFE to length remains significantly dependent on sequence size (data not shown).

### A simple correction of the normalization procedure can substantially remove any intrinsic dependence of the MFE on sequence length in RNAs longer than 40 nucleotides

**Figure 5:**
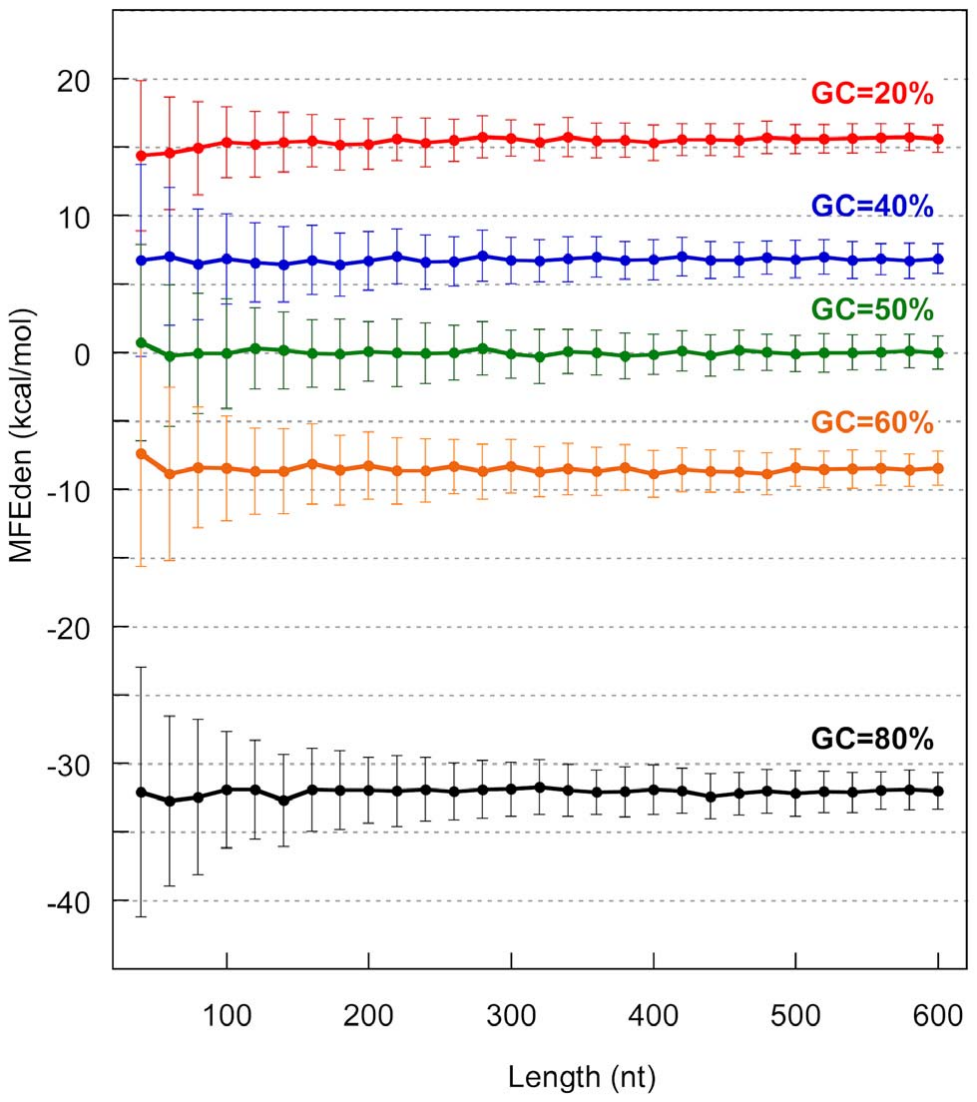
MFEden versus length. Plot of the mean MFEden versus length for shuffled sequences with GC-content of 20%, 40%, 50%, 60% and 80%, and for sequence lengths ranging between 40 and 600 nt with steps of 20 nt. Each point corresponds to the mean value of 100 shufflings. The lines connect MFEden values with the same GC-content. Vertical lines indicate standard deviation (N = 100).

**Figure 6:**
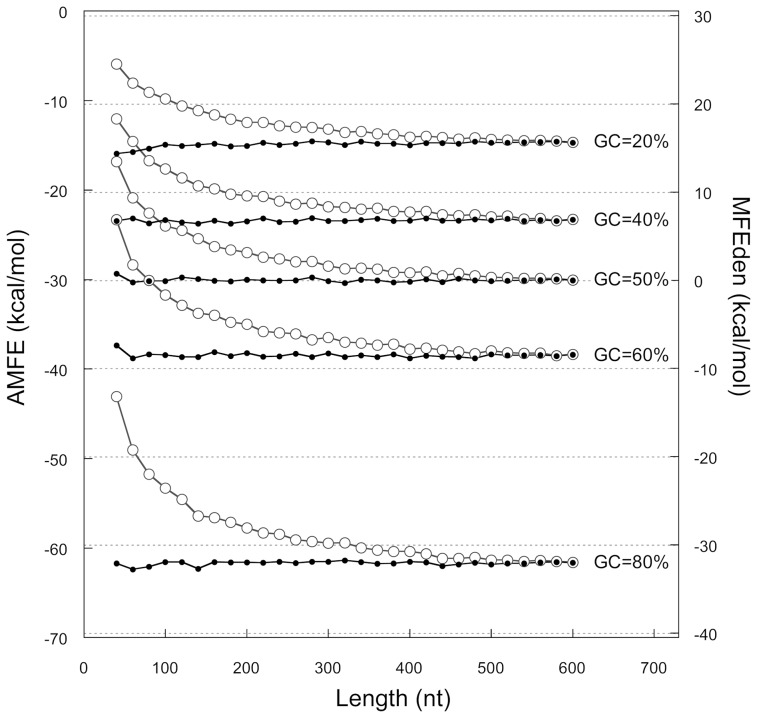
MFEden and AMFE versus length. Comparison of MFEden (black points) and AMFE (grey open circles) for shuffled sequences with GC-content of 20%, 40%, 50%, 60% and 80%, and for sequence lengths ranging between 40 and 600 nt with steps of 20 nt. Each point corresponds to the mean value of 100 shufflings. The lines connect values with the same GC-content.

Our results show that the AMFE bias is generated by the combined effect of two causes: the non-perfect linearity of the MFE with sequence length and an inaccurate mathematical procedure that does not take into account that the regression line of the MFE versus the length does not intersect the axes’ origin. Here, we introduce a new length-normalized MFE index, termed MFEden, which is computed to reduce the effects of the two causes of AMFE bias:



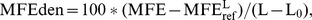
where L is the length (number of nucleotides) of the analyzed sequence, MFE_ref_^L^ is the precalculated average MFE computed for a shuffled sample containing L nucleotides and an equimolar ratio of the four nucleotides, and L_0_ is a predefined optimal constant amount that shifts the MFE-versus-length regression line to the origin of the graph. Figure 5 shows the plot of the mean MFEden versus length for shuffled sequences with GC-content of 20%, 40%, 50%, 60% and 80% and for sequence lengths ranging between 40 and 600 nt. The large decrease of AMFE bias in the corrected index MFEden is evident in Figure 6 where the two indices are directly compared. As shown, in the critical range of length, where the bias makes AMFE impractical (between approximately 40 and 300 nt), the MFEden is unaffected by length.

### The information content of the MFEden

**Figure 7:**
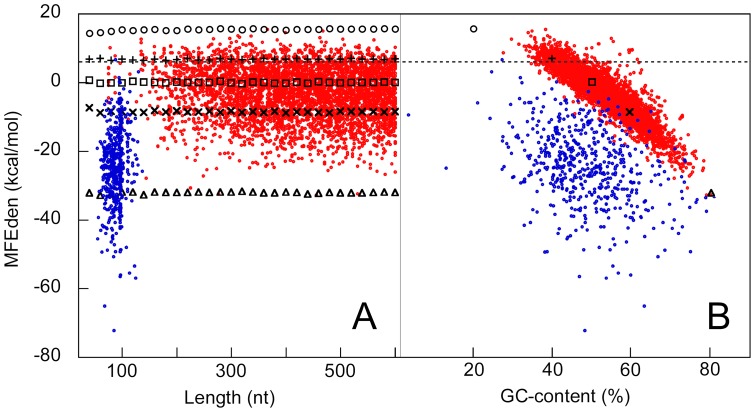
MFEden of human CDSs and pre-miRNA. MFEden of CDSs (red circles) and pre-miRNA (blue circles) are plotted versus sequence length (panel A) and GC-content (panel B). Black symbols indicate the mean MFEden values computed from shuffled sequences: GC-content: 20% (circle), 40% (plus), 50% (square), 60% (×), and 80% (triangle). A horizontal broken line indicates the MFEden level expected for the genomic GC-content.

The MFE of an RNA sequence is determined by the combined contributions of its length, nucleotide content and nucleotide order. MFEden excludes the component of free energy associated with sequence length but includes those related to nucleotide order and composition, also indirectly giving an estimate of their relative contributions. To illustrate the information content of MFEden, here we report an analysis using high confidence sets of two human RNA families: coding sequences (CDSs) and micro RNA precursors (pre-miRNAs) (see Materials and Methods). The panels A and B in Figure 7 show the scatterplot of the MFEden of the CDSs (red circles) and pre-miRNAs (blue circles) versus the sequence length and the GC-content, respectively. As shown in Figure 7, in agreement with the results previously reported [3], pre-miRNAs are characterized by an MFEden lower than expected according to their nucleotide content. The MFEden of the coding sequences is approximately that expected for our shuffled sequences with a comparable GC-content. Moreover, the MFEden of the coding sequences appears to be scarcely affected by sequence length (Figure 7A), indicating that free energy density, on average, changes little from short to long (<600 nt) CDSs. From the human genomic GC-content, which is approximately 40.9% [18], we estimated the MFEden expected for a typical genomic sequence equal to about 6.2 kcal/mol. This estimated MFEden level is very close to 5.3 kcal/mol, which is the average MFEden that we computed for a sample of 100 genomic sequences, 100 nt-long, randomly chosen inside each human chromosome (2400 sequences in all). In Figure 7B, the estimated level of MFEden for a typical genomic sequence is indicated by an horizontal broken line showing the different nature of the MFE density in CDSs and pre-miRNAs. CDSs on average exhibit more negative MFEden than expected for the genomic GC-content. The folding stability of the CDSs is very close to that expected for their own GC-content, suggesting a very weak role of nucleotide order in determining their low free energy density. In contrast with CDSs, Figure 7B shows that, although the GC-content also contributes significantly to the high folding stability of pre-miRNAs, for this functional RNA family the nucleotide order plays a dominant role in determining its large stability with respect to a typical genomic sequence.

### MFEden analysis of human functional RNAs

Along with CDSs and pre-miRNAs, we analyzed the MFEden of functional RNA sequences ranging between 40 and 600 nt. The datasets used in this study contain the most frequent families of human functional RNAs stored in the Rfam.fasta file of the Rfam database [19] and the sequences of small nucleolar RNAs (snoRNA) H/ACA and C/D box downloaded from the snoRNABase [20].

We found that the average length of each RNA family is not significantly correlated with its average MFEden (Pearson correlation coefficient (R_p_) =  −0.1531, N = 13, p = 0.6176), indicating that sequence length does not appear to be significantly constrained by folding free energy demands. We roughly estimated the contribution of nucleotide composition to the MFEden of each RNA by the mean MFEden of our shuffled sequences with the corresponding GC-content. The contribution of sequence order was valued by subtracting the estimated contribution of nucleotide composition from the computed MFEden. The average contributions to MFEden of the two sequence properties are, in each RNA family, positively correlated (R_p_ = 0.6689, N = 13, p<0.02), suggesting that sequence composition and nucleotide order, in contrast with sequence length, concur to determine the level of the thermodynamic stability that characterizes a functional RNA family.

**Figure 8:**
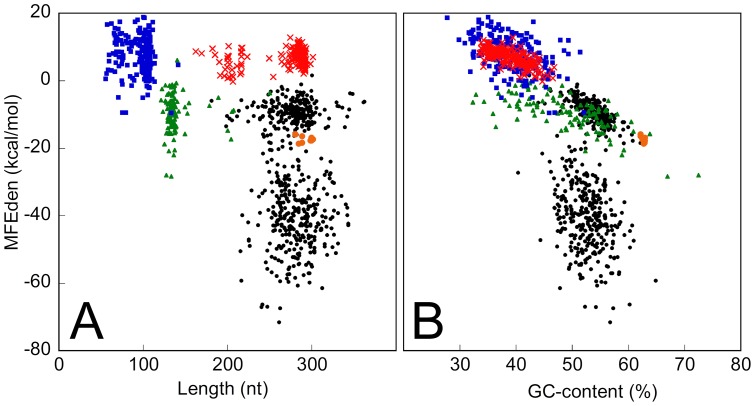
MFEden of human RNA families. The MFEden of the functional RNA families SRP RNAs (black points), U6 snRNAs (blue squares), RSV RNAs (red Xs), and H/ACA box RNAs (green triangles) plotted versus the sequence length (panel A) and the GC-content (panel B). Orange points indicate the 17 human SRP seed sequences of Rfam database.

**Figure 9:**
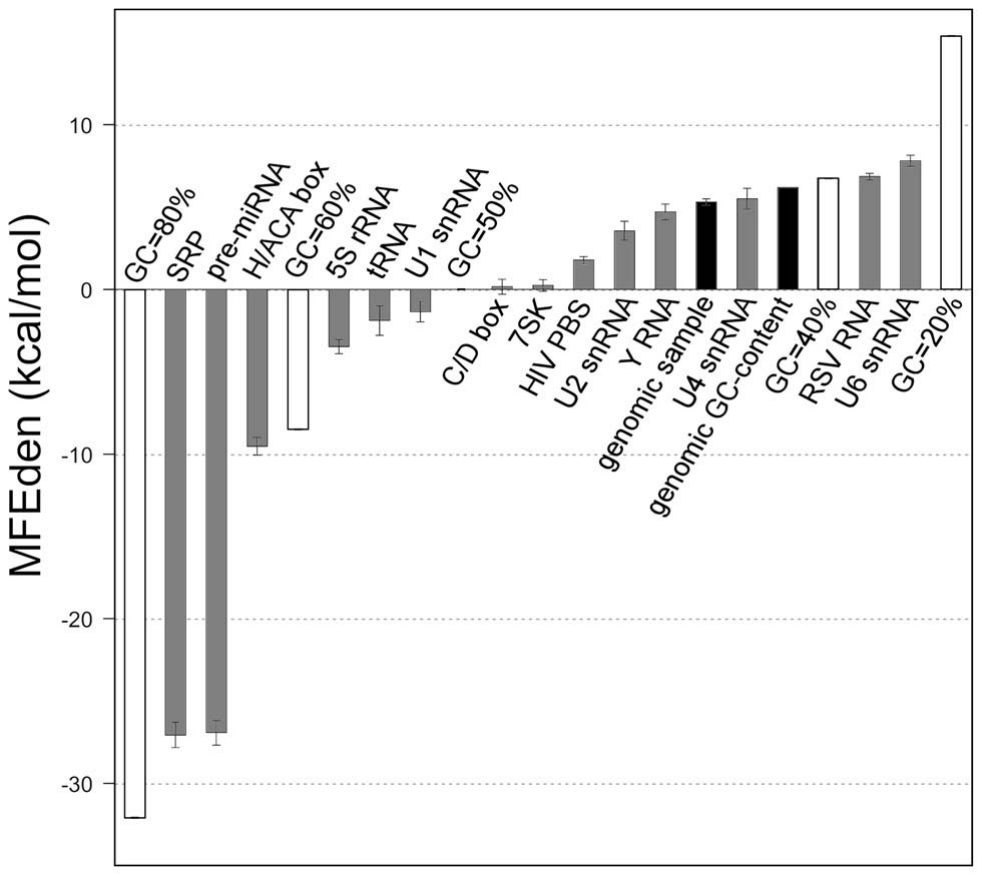
MFEden of 14 human functional RNA families. Bar plot showing the mean MFEden of 14 human functional RNA families (grey bars) compared with the mean MFEden of shuffled sequences with GC-content equal to 20%, 40%, 50%, 60% and 80% (white bars), the mean MFEden of 2400, 100 nt-long, genomic sequences taken at random and the MFEden expected for the genomic GC-content (black bars). The vertical bars indicate the standard errors of the means.

The results of our analysis also show that each RNA family is characterized by a restricted and well-defined combination of MFEden, length and GC-content. As an example, Figure 8 reports the MFEden of signal recognition particle RNAs (SRP RNAs), U6 spliceosomal RNAs (U6 snRNAs), Rous sarcoma virus RNAs (RSV RNAs), and H/ACA box RNAs plotted versus the sequence length (panel A) and the GC-content (panel B). In general, most of the RNA families examined here exhibit an average free energy density more negative than expected for a typical genomic sequence (Figure 9). In particular, SRP and H/ACA box RNAs and pre-miRNAs, exhibit the most negative average free energy density. Only small nuclear ribonucleic acids (snRNA) U4 and U6 and Rous sarcoma virus (RSV) RNAs have an average free energy density equal or slightly more positive than that expected for the genomic sequences. The case of the SRP family sequences stored in the Rfam database is interesting. The MFEden (and MFE) distribution of the SRP RNAs is bimodal, defining two distinct ranges of MFE density that are characterized by a similar range of GC-content (Figure 8). Moreover, surprisingly, the 17 human SRP seed sequences (orange points in Figure 8), which are used as high-quality reference RNAs for predicting SRP sequences stored in the Rfam database, exhibit a GC-content higher than that of the 99% of the SRP sequences in the database (Figure 8).

## Discussion

MFE divided by the number of nucleotides is usually defined as length-normalized MFE [2], [6]–[8], [21]–[24]. Strictly speaking, it should mean that, using the normalized MFE indices, the differences in the minimum free energies of RNA molecules can be almost exclusively attributed to their nucleotide order and composition, regardless of their lengths. In fact, the length-normalized index AMFE was introduced by specifying that, after the MFE is adjusted, sequences with lengths ranging from 60 to 400 nt are comparable based on their MFEs [9]. Accordingly, normalized MFE has been used because MFE values are strongly correlated with length [21], and because it serves as a comparable measure without excessively penalizing the shorter precursor microRNAs or favouring the longer mRNAs [24]. Similarly, it has been reported that normalization renders the MFE of hairpins of different lengths comparable [6], and normalized MFE was used to analyze the relationship between folding free energy and GC-content in mRNA sequences with different lengths [2].

Normalized MFEs have been employed for various purposes. For example, normalization was used to improve structure prediction by discarding segments whose normalized equilibrium free energies were smaller than a threshold value [7]. Normalized minimum free energy was also used to compare evolutionary relationships between micro-RNA genes and their functions [8], and its usefulness in identifying new non-coding RNAs was compared with other measures [4]. AMFE helped to find thermodynamics differences between nuclear-encoded microRNAs localized principally in mitochondria and cytosol [25]. Normalized MFE was also used in the search for distinctive criteria to predicting authentic precursors of microRNAs [22], [24], for comparing thermodynamic stability [26], and to improve algorithms for RNA folding predictions [23], [27], [28].

Despite the significant number of works using normalized MFE, to our knowledge, the linearity of the relationship between MFE and sequence length, as well as the dependence of normalized MFEs on RNA size, has not been thoroughly tested and lacks quantitative substantiation. Here, we show that MFE does not decrease linearly with sequence length, especially in the range of sequences shorter than 100 nt. This deviation from a perfect linear relationship, along with the bias introduced by dividing the MFE by the length of the sequence, makes the normalized MFE of differently sized RNA sequences not directly comparable. In fact, we found that the magnitude of AMFE bias associated with length is comparable to the AMFE variation associated with the GC-content and with the variability produced by the random arrangement of nucleotides in the sequence. We also found that stacking and hairpin loop interactions are responsible for almost all the AMFE bias. The AMFE bias is higher in shorter RNAs and makes the AMFE index unsuitable for sequences shorter than approximately 300 nt. To extend the applicability of normalized MFEs to sequences shorter than 300 nt, we introduce a new index, called MFEden, obtained by a simple correction of the AMFE formula. The new MFEden index extends the applicability of AMFE to RNA longer than 40 nt. This is a big improvement if we consider that, of the 2208 functional RNA families stored in the Rfam database, 2023 (92%) have an average length ranging between 40 and 300 nt, and overall, 2104 families (95%) have an average length longer than 40 nt.

The stability of an RNA sequence is determined by the combined contributions of its length, nucleotide content and nucleotide order. In other words, if the local or the overall folding thermodynamic stability is important for the correct functionality of an RNA, it can be reached by acting on these three structural elements. Depending on the specific RNA function, these three elements could be differently constrained, and the evaluation of their respective contributions to the overall free energy can be useful for their assignment to a functional class. From the perspective of free energy components associated with the three structural elements, the MFE, Z-score [29] and MFEden represent very different indices. The MFE of an RNA includes the free energy components of all three structural elements: sequence length, nucleotide content and nucleotide order. The Z-score represents a different method for quantifying the RNA secondary structure stability [29]. This index measures the distance between the MFE of the analyzed RNA sequence and the average MFE of a number of sequences generated by the random permutation of its nucleotides. The distance is measured in terms of the SD of the permutated sequences and, since the Z-score is a dimensionless index, lacks a direct relationship with the absolute amount of the free energy involved in folding stability. Because the shuffled sequences used as reference contain the same composition and the same number of nucleotides of the analyzed sequence, the Z-score index measures only the component of folding energy associated with the order of nucleotides in the sequence. This important point should be considered when the Z-score of two sequences is compared. In fact, for example, two RNAs with the same length and Z-score can differ significantly in their thermodynamic stability due to different GC-content. In addition, calculating the Z-score, especially for analyzing large RNA families, is laborious and time-consuming because of the sequence randomization procedures and the MFE computation of all simulated sequences. Differently from the MFE and the Z-score, the MFEden excludes the free energy contribution associated with the sequence length but includes the components related to nucleotide order and composition, also, indirectly, providing a rough estimate of their relative contributions. Moreover, the MFEden is measured in free energy units, its computation is not laborious and time-consuming, and it is suitable for large datasets. The MFEden analysis of the human RNA families examined in this work suggests that the GC-content and the nucleotide arrangement generally concur to determine the level of the thermodynamic stability that characterizes a functional RNA family, whereas the sequence length does not appear to be significantly constrained by folding free energy demands. This lack of correlation between the MFEden and the RNA size suggests that sequence length is scarcely informative about the folding stability demands of an RNA family, and therefore represents a confusing variable when the MFE of different RNA families is compared. For this reason, MFE density appears more informative than MFE about the thermodynamic stability requirements of an RNA family. Accordingly, for example, U6 spliceosomal RNA family is characterized by a significantly high MFEden (Figures 8 and 9) that is consistent with its biological function. Such a low structure stability should facilitate the large conformational changes that U6 RNAs experience during the assembly of the spliceosome [30]. Conversely, pre-miRNA family is characterized by a significantly low MFEden (Figures 7 and 9). This high global structural stability is compatible with the necessity of pre-miRNA to maintain the stem-loop structure that is recognized and cleaved by double-stranded specific nucleases (Dicer family) by a process that is critical for the miRNA biogenesis [31]. The lack of correlation between the MFEden and the RNA size also suggests that the intrinsic higher stability of longer sequences is generally not compensated by a low level of GC-content or a decreased amount of stacking interactions, suggesting that there is not a general optimal level of thermodynamic stability at which every RNA tends. We also found that each RNA family is characterized by a restricted and well-defined combination of MFEden, length and GC-content. Furthermore, pre-miRNA, SRP and ACA_box RNAs exhibit significant negative MFE densities than the other RNAs and the typical genomic sequences. These differences in the MFE density of the RNA families can be exploited to improve the accuracy of sequence filtering for predicting non-coding RNAs.

In conclusion, this work demonstrates that length-normalized indices of MFE are biased measures of folding free energy density and proposes a new index with improved applicability for short RNA sequences. Unlike the Z-score, the new index, termed the MFEden, is simple and not time-consuming to compute, suitable for large datasets, and includes the folding free energy component associated with GC-content. An analysis of the MFEden of real sequences shows the different roles of length, GC-content and nucleotide order in the folding stability of RNA families and suggests the possible use of the MFEden to improve algorithms for predicting new RNAs or for their assignment to a functional class.

## Materials and Methods

### Data processing and analysis

All data were processed using software programs developed in our laboratory in the C# language that were tested by independent computational tools and manual calculations. Our software also includes programs to randomly shuffle the nucleotides of RNA sequences using the Fisher-Yates algorithm [32] and to read genomic sequences in a random position in the chromosomes. Statistical analysis was performed using STATISTICA (version 8.0, Statsoft, Inc.).

### Computation of MFE, AMFE and MFEden

MFE was computed using two programs: RNAfold, included in the ViennaRNA software package version 2.1.5 [15], [16]; and Quickfold, from the Mfold web server (http://mfold.rna.albany.edu/?q=DINAMelt/Quickfold) [13], [14]. For very short sequences, we found that the MFEs computed by Quickfold (Mfold) were sometimes positive. In these cases, global free energy were set to 0 kcal/mol.

AMFE was calculated by dividing MFE by the sequence length and then multiplying the result by 100 to relate the index to a 100-nucleotides segment: AMFE = 100•MFE/length [9].

MFEden was computed using the formula MFEden  = 100*(MFE-MFE_ref_^L^)/(L-L_0_), where MFE_ref_^L^ is the expected MFE for a sequence with L nucleotides and equimolar ratios of A, C, G and U nucleotides. The expected MFEs were estimated by the mean MFE of 2000 random shufflings of sequences from a set with increasing lengths: from 40 to 600 nt, in steps of 4 nt. The estimated MFE of intermediate lengths were linearly interpolated (see Dataset S1 for MFEs computed by RNAfold). The optimal L_0_ value for MFEs computed by RNAfold was determined empirically equal to 8 nt.

In all simulated sequences, including those with various GC-content, Watson and Crick complementary bases were present at the same frequency: number of As equal to number of Us, and number of Cs equal to number of Gs.

### Human RNA sequences

All native sequences analyzed in this work were included in the taxonomic category of Homo Sapiens. RNA coding sequences were downloaded from the consensus CDS database (CCDS) (release 15) (ftp://ftp.ncbi.nlm.nih.gov/pub/CCDS/) [33], which provides high-quality human CDS data. Of the 29064 high-quality sequences downloaded from the CCDS database, we used the 4379 sequences with length included between 40 and 600 nt.

From the miRBase database (ftp://mirbase.org/pub/mirbase) (release 20) we downloaded the set of high-confidence microRNAs [34] which includes 278 human sequences.

The human most frequent families in the Rfam.fasta file stored in the Rfam database [19] (release 11.0) (ftp://ftp.ebi.ac.uk/pub/databases/Rfam/) were used in this study: 5S ribosomal RNAs (ID: RF00001), U1 spliceosomal RNAs (ID: RF00003), U2 spliceosomal RNAs (ID: RF00004), Transfer RNAs (ID: RF00005), U4 spliceosomal RNAs (ID: RF00015), Signal recognition particle RNAs (ID: RF00017), Y RNAs (ID: RF00019), U6 spliceosomal RNAs (ID: RF00026), 7SK RNAs (ID: RF00100) and Rous sarcoma virus RNAs (ID: RF01417).

The sequences of human H/ACA and C/D box small nucleolar RNAs were downloaded from the snoRNABase [20].

### Estimate of the MFE density components associated with GC-content and nucleotide order

The expected values of MFEden for a specific GC-content was estimated by a polynomial interpolation of MFEden reference data computed for shuffled sequences with varying levels of GC-content (20%, 40%, 50%, 60% and 80%). The approximate MFEden component associated with GC-content was estimated by subtracting the MFEden expected for the genomic GC-content from the MFEden expected for the GC-content of the sequence analyzed. The estimate of the MFEden associated with nucleotide order was performed by subtracting the MFEden expected for the GC-content from the overall computed MFEden of the analyzed RNA.

## Supporting Information

Dataset S1 **RNAfold precalculated estimate of MFE expected for RNA sequences with L nucleotides and equimolar ratios of A, C, G and U.** The expected MFEs were estimated by the mean MFE of 2000 random shufflings of sequences from a set with increasing lengths: from 40 to 600 nt, in steps of 4 nt. The estimated MFE of intermediate lengths were linearly interpolated.
(TXT)
